# Supplementary material for: On the accuracy of image registration in portable low-field 3D brain MRI
Source: Res Sq. 2026 Apr 6:rs.3.rs-8255109. Preprint. [Version 1] doi: 10.21203/rs.3.rs-8255109/v1 (PMC13082117; doi:10.21203/rs.3.rs-8255109/v1)
Supplement: Supplement 1 [file NIHPPRS8255109V1-supplement-1.pdf]

Supplementary Figures

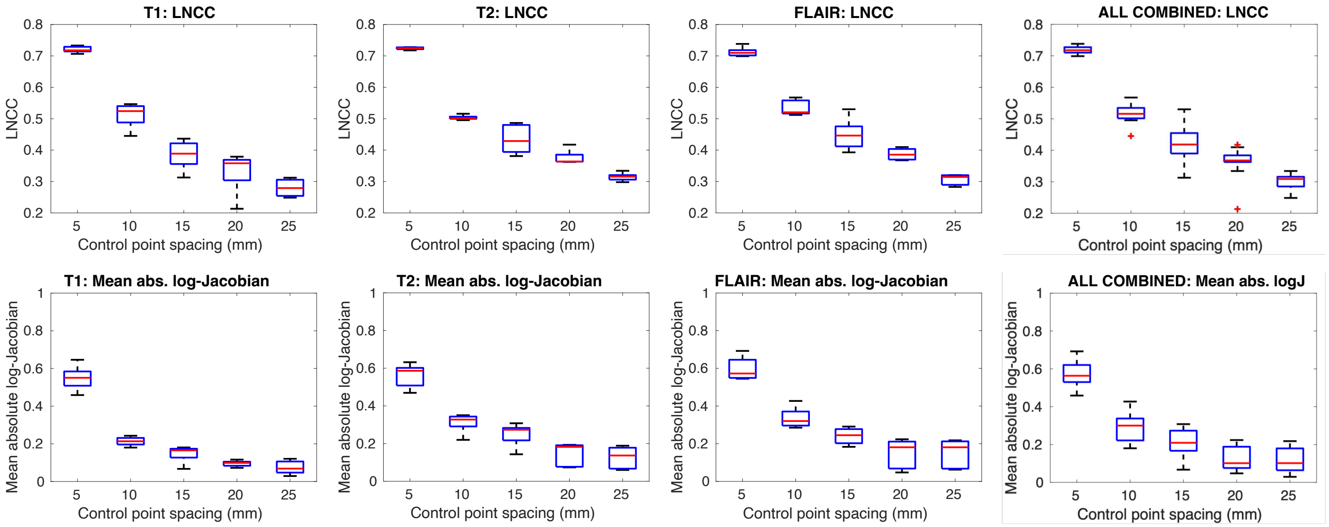

Figure S1. Box plots for LNCC and mean absolute log-Jacobian for phantom registration from high to low field, over five different low-field sessions (details in Methods), for five different control point spacings of the B-Spline transform in NiftyReg. We show plots for T1, T2, and FLAIR scans, as well as for all pulse sequences combined. The boxes show medians (in red), interquartile ranges (IQR, in blue), whiskers ($\pm 1.5 \times$IQR, in black), and outliers (red crosses). We selected 20 mm as a compromise, since smaller spacings led to rapidly increasing irregularity, and the 20 mm transform sufficed to achieve accurate visual alignment (Figure 2d), i.e., higher LNCC values seem to indicate overfitting.
